# Clinical characteristics of epithelioid hemangioendothelioma: a single-center retrospective study

**DOI:** 10.1186/s40001-019-0375-8

**Published:** 2019-02-28

**Authors:** Xin Wu, Binglu Li, Chaoji Zheng, Tao Hong, Xiaodong He

**Affiliations:** Department of General Surgery, Peking Union Medical College Hospital, Chinese Academy of Medical Sciences and Peking Union Medical College, No. 1 Shuaifuyuan, Dongcheng District, Beijing, 100730 China

**Keywords:** Epithelioid hemangioendothelioma, Vascular tumor, Treatment, Prognosis, Retrospective analysis

## Abstract

**Background:**

Epithelioid hemangioendothelioma (EHE) is a rare malignant vascular neoplasm with increasing incidence. However, its clinical characteristics remain unclear due to its low incidence. This study aimed to investigate the features of EHE.

**Methods:**

Patients with EHE treated at our institution between January 2000 and June 2018 were enrolled. Data including demographic characteristics, treatment patterns, pathological results, and prognosis were collected, and a retrospective database was constructed for analysis.

**Results:**

The cohort comprised 33 patients with a mean age of 48.0 ± 16.0 years. Eighteen (54.5%) patients were asymptomatic. The laboratory tests were unremarkable except in five and two patients who had increased CA 125 and CA 19-9, respectively. Twenty-one patients underwent surgery, while 12 patients underwent only biopsy. The postoperative morbidity rate was 28.6% (6/21). The anatomical sites of the primary lesions varied. Immunohistochemical staining was positive for CD34 and CD31 in most patients. Twenty-six patients (78.8%) were followed up at a range of 1–201 months, and 6 patients died during this period. The 1-, 3-, and 5-year cumulative survival rates were 96.2%, 87.0%, and 75.3%, respectively. The patients who had metastases or only underwent biopsy showed significantly higher mortality.

**Conclusions:**

EHE is a rare malignant vascular tumor that can occur in any site of the body. Surgery is the primary choice of treatment, and pathologic evaluation is the gold standard for diagnosis. Metastases and unresectability are associated with poor prognosis.

## Background

Epithelioid hemangioendothelioma (EHE) is a rare malignant vascular neoplasm that arises from vascular pre-endothelial or endothelial cells [1]. It can occur anywhere in the body, such as the liver, lung, skin, bone, spleen, pleura, and lymph nodes [2–4]. The first case of EHE was reported by Weiss and Enzinger in 1982 [5]. In the 2013 World Health Organization classification of sarcomas, EHE is distinguished from other vascular tumors and defined as an independent disease [6]. EHE shows low- to intermediate-grade malignancy, and its clinical behavior is more indolent than angiosarcoma. EHE is extremely rare, with an incidence of 1 in 1 million [1], and the literature is limited to case reports and few retrospective case series with small samples. As such, the clinical manifestations, pathologic characteristics, surgical patterns, and prognoses for EHE remain unclear.

This study aimed to analyze the clinical features of EHE and to explore the diagnostic and treatment patterns. Toward this goal, we compared and analyzed the differences between survival and death during the follow-up period.

## Methods

### Patients

We retrospectively assessed patients with EHE in a Han Chinese Population who were diagnosed and treated at Peking Union Medical College Hospital between January 2000 and June 2018. All the medical records were studied systematically by two independent doctors. Patients were selected based on the following criteria: pathological diagnosis of EHE via surgery or biopsy and complete medical records. Patients with hemangioma, hemangiosarcoma, and other subtypes of hemangioendothelioma (HE) (Dabska tumor, retiform HE, kaposiform HE, pseudomyogenic HE, and composite HE) were excluded. Discordance in the reviews of the medical records was resolved through a discussion.

Data, including demographic characteristics, clinical symptoms, test results, treatment patterns, pathological results, and prognosis, were collected from both outpatient and inpatient medical records, and a retrospective database was constructed for the analysis. This study was approved by the Peking Union Medical College Hospital Institutional Review Board, and the need for informed consent was waived due to the retrospective nature of the study.

### Treatment

The feasibility of surgery was assessed via preoperative imaging tests. Patients with extensive intrahepatic lesions or distant metastases were considered to have unresectable disease, and intractable cases were managed through multidisciplinary consultation. The patients with resectable EHE underwent surgery. Frozen section was used to ensure negative margin, and diagnoses were confirmed via postoperative pathology. Meanwhile, patients with unresectable primary lesion or distant metastases were diagnosed via biopsy. Complications were defined as any adverse event occurring within 30 days after surgery or biopsy. Outpatient interviews, telephone calls, and e-mails were used for follow-up. Computed tomography (CT) or ultrasound was performed every 6 months during the first 2 years and annually thereafter.

### Statistical analysis

The study endpoints were death or patient status on last follow-up (October 2018).

Statistical analysis was performed by an independent statistician. Linear variables were described using mean ± standard deviation, while categorical variables were presented as absolute number or frequency. Differences between groups were analyzed using Fisher’s exact test or Student’s *t* test as appropriate. Survival probability was estimated using the Kaplan–Meier method with log-rank test. All statistical analyses were conducted using Statistical Package for Social Sciences software (SPSS, version 19.0, Chicago, IL, USA), and a *p* value of < 0.05 was considered statistically significant.

## Results

### Clinical manifestations

**Fig. 1:**
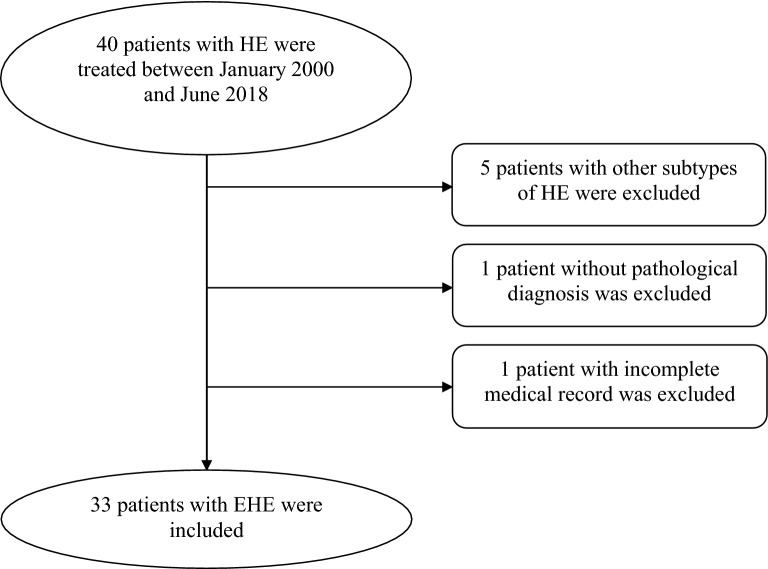
Flow diagram of patient selection. *HE* hemangioendothelioma, *EHE* epithelioid hemangioendothelioma

Of the 40 patients with HE treated at our institution, 33 were included in the analysis (Fig. 1). The demographic data and preoperative signs and symptoms are presented in Table 1. There were 12 male patients (36.4%), and the male-to-female incidence ratio was 1:1.75. Eighteen (54.5%) patients were asymptomatic and were diagnosed with a space-occupying lesion either incidentally or during routine physical examination. Except for one patient with rectal adenocarcinoma, the remaining patients had no coexisting tumor.

**Table 1:** Demographic data and preoperative signs and symptoms in patients with EHE

Characteristics	*n* (%)
Gender	
Male	12 (36.4%)
Female	21 (63.6%)
Age (years)	48.0 ± 16.0
Range	18–77
Positive signs	
None	18 (54.5%)
Palpable tumors	8 (24.2%)
Local tenderness	3 (9.1%)
Skin damage	2 (6.1%)
Thoracic deformity	1 (3.0%)
Muffled heart sounds	1 (3.0%)
Symptoms	
None	18 (54.5%)
Backache	3 (9.1%)
Stuffy chest	2 (6.1%)
Nasal congestion	2 (6.1%)
Ostealgia	2 (6.1%)
Hemoptysis	1 (3.0%)
Ear bleeding	1 (3.0%)
Weakness	1 (3.0%)
Fever	1 (3.0%)
Leg swelling	1 (3.0%)
Colporrhagia	1 (3.0%)

*EHE* epithelioid hemangioendothelioma

### Laboratory examination and imaging

All patients underwent laboratory tests including complete blood count, coagulation function, and tumor marker screening. The results of the complete blood count and coagulation function test were unremarkable. However, CA 125 levels were elevated in five patients (38.3 U/mL, 49.7 U/mL, 52.5 U/mL, 131.7 U/mL, and 1436 U/mL; reference level, 0–35.0 U/mL), and CA 19-9 levels were elevated in two patients (44.0 U/mL and 356.5 U/mL; reference level, 0–37.0 U/mL). Ultrasonography was performed in 24 patients and revealed mixed echo or hypoechoic lesion with clear or unclear boundary. CT was performed in 19 patients and showed low- or mixed-density tumors that might have delayed enhancement. Magnetic resonance imaging (MRI) was performed in six patients and revealed a space-occupying lesion with low–middle signal intensity in T1-weighted imaging and high signal intensity in T2-weighted imaging. Overall, the imaging characteristics were relatively nonspecific.

### Treatment patterns

Twenty-one patients underwent surgery. Within them, two underwent local radiotherapy after scalp mass resection, and one patient who had comorbid rectal adenocarcinoma underwent chemotherapy for the rectal cancer. All the other patients received no adjuvant therapy. There were 12 patients who underwent biopsy only due to unresectable primary lesion or distant metastases. Of them, six were administered chemotherapy, while two underwent radiotherapy. Postoperative complications occurred in six patients and included fever (*n* = 2), wound infection (*n* = 1), wound inflammation (*n* = 1), urinary retention (*n* = 1), and intra-abdominal hemorrhage (*n* = 1). Fever, wound inflammation, and urinary retention were managed using antipyretics, wound dressing, and catheterization, respectively. Wound infection was treated via reoperation. The patient who developed intra-abdominal hemorrhage had severe underlying disease and died after splenectomy. In total, four, one, and one patients were classified into grade I, grade IIIb, and grade V complication following the Clavien–Dindo classification of surgical complications [7]. The postoperative morbidity rate was 28.6% (6/21).

### Pathology

**Fig. 2:**
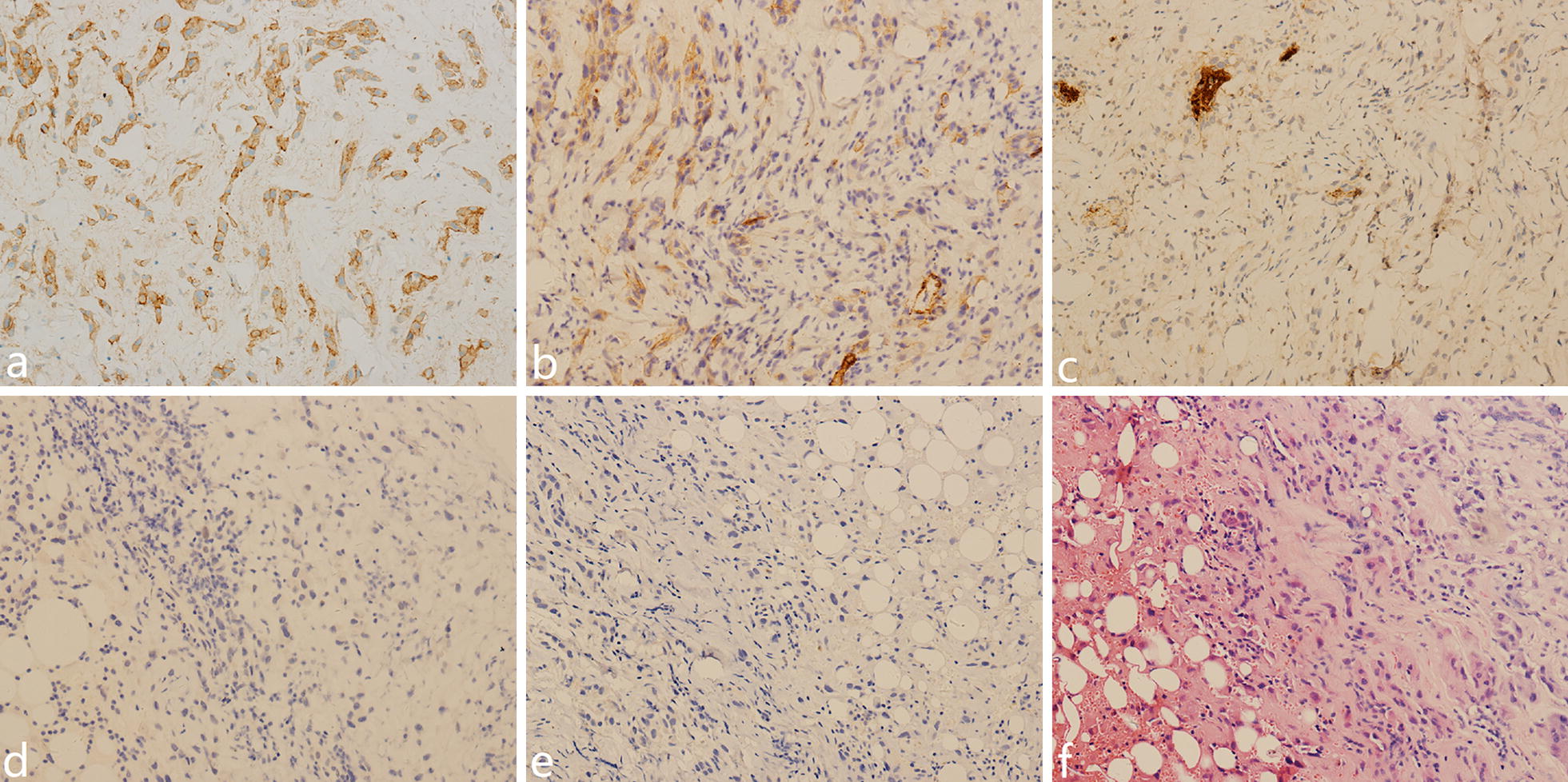
Microscopic immunohistochemistry findings (magnification ×150). Immunohistochemically, the cells stain positively for CD 31 (**a**), CD 34 (**b**), and F8-R (**c**), and negatively for AE1/AE3 (**d**), and SMA (**e**). These characteristics combined with hematoxylin and eosin image (**f**) reveal EHE. *EHE* epithelioid hemangioendothelioma

All patients were pathologically diagnosed with EHE (Fig. 2). The anatomical sites of the primary lesions are shown in Table 2. The most common sites of the primary tumor were the liver, scalp, and spine. The mean tumor diameter was 5.8 ± 2.9 cm (range, 0.6–10.7 cm). Immunohistochemical staining was performed for all patients. The most common positive markers were CD34 (32/33, 97.0%) and CD31 (32/33, 97.0%), followed by vimentin (14/16, 87.5%) and F8-R (16/19, 84.2%).

**Table 2:** Anatomical sites of the primary lesions in patients with EHE

Anatomical site	Number (*n*)
Liver	4
Scalp	4
Spine	4
Nasal cavity	2
Spleen	2
Chest wall	2
Lung	2
Groin	1
Mastoid process	1
Skull	1
Tongue	1
Neck	1
Esophagus	1
Axilla	1
Atrium	1
Mediastinum	1
Small intestine	1
Presacral area	1
Cervix uteri	1
Hip	1

*EHE* epithelioid hemangioendothelioma

### Follow-up

**Fig. 3:**
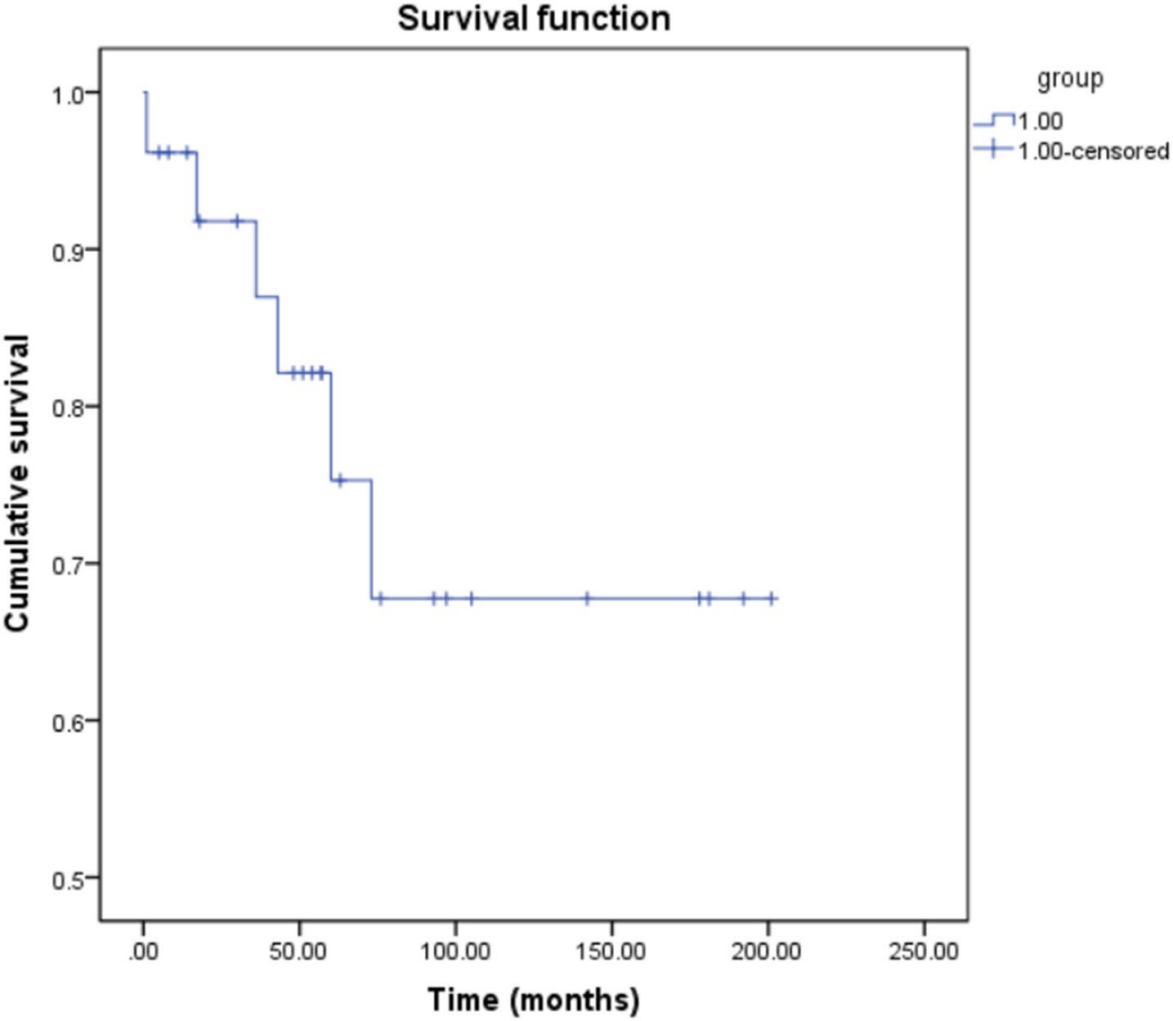
Kaplan–Meier curve of cumulative survival rate of patients with EHE (*n* = 26). The 1-, 3-, and 5-year cumulative survival rates of patients with EHE were 96.2%, 87.0%, and 75.3%, respectively. *EHE* epithelioid hemangioendothelioma

Twenty-six patients (78.8%) were followed up at a mean period of 73.1 ± 60.0 months (range, 1–201 months). Six patients died during the follow-up period. One patient died from intra-abdominal hemorrhage after splenectomy, while five died from tumor-related diseases. The cumulative survival rates for the followed-up patients are shown in a Kaplan–Meier curve (Fig. 3). The 1-, 3-, and 5-year cumulative survival rates were 96.2, 87.0, and 75.3%, respectively. The 26 patients were further divided into two groups based on the follow-up results: the survivors (*n* = 20) and non-survivors (*n* = 6). There were no significant differences in sex distribution and mean age between the two groups. However, the patients who had metastases or underwent biopsy only showed significantly higher mortality than those who underwent surgery (Table 3).

**Table 3:** Comparison of clinical features between survivors and non-survivors

	Total (*n* = 26)	Survivors (*n* = 20)	Non-survivors (*n* = 6)	*p* value
Male/female (*n*)	8/18	6/14	2/4	1.000
Age (years)	48.7 ± 16.0	47.0 ± 15.7	54.7 ± 17.1	0.311
Metastases (*n*)	10	4	6	0.001
Surgery/biopsy (*n*)	16/10	15/5	1/5	0.018

## Discussion

EHE is more frequent in women than in men at a 4:1 ratio, and the median age of disease onset is 36 years [1, 8]. It can present in numerous primary sites and manifest as heterogeneous symptoms. Most patients have no specific symptoms and are diagnosed incidentally. In the present study, EHE was more common in women than in men at a 1.75:1 ratio, and the mean age of onset was 48.0 ± 16.0 years. There were more male patients and elderly in the present study than that reported in the literature. The signs and symptoms varied, and more than 50% of the patients were asymptomatic. In symptomatic patients, the symptoms were nonspecific and limited only to the site of involvement. These factors made the diagnosis of EHE difficult.

There are no specific laboratory tests for EHE. Complete blood count and coagulation function are rarely affected by EHE, and even tumor markers only abnormally increase in few patients [9–11]. In the present study, only five and two patients (15.2%) had abnormal CA 125 and CA 19-9, respectively. Meanwhile, imaging can be valuable for diagnosis [4]. Tumors can appear as low-density images and show mild delayed enhancement in CT. EHE is more frequently observed in soft tissues than visceral organs, and an isolated lesion is quite common [12, 13]. MRI is more diagnostic for soft tissue tumors and more useful for evaluating tumor resectability.

Because EHE is a rare malignancy that can occur in any organ systems, no optimal treatment strategy has been established. If the lesion is localized, surgical resection is the primary treatment modality [4], and it also allows for diagnosis and treatment in a single procedure. By contrast, if complete resection is not possible, biopsy is required for pathological diagnosis.

Retaining organ function while aiming for negative margins is crucial in surgery. If curative surgery cannot be achieved, the appropriate adjuvant therapy should be considered. Radiotherapy is suitable for patients with lesions involving the bones [14, 15], while chemotherapy is suitable for patients with advanced tumor or lesions located in deep tissues [16, 17]. However, EHE is generally not sensitive to either radiotherapy or chemotherapy, and the standard adjuvant therapy is still controversial. Targeted therapy is also widely used in EHE. Several drugs such as pazopanib, sorafenib, and bevacizumab have been reported to have a therapeutic effect on EHE [18–20]. For patients with unresectable hepatic EHE, liver transplantation could be considered [21]. In asymptomatic patients with diffuse lesions, a “wait and see” management may be possible as spontaneous regressions have been reported [22].

Pathology is the gold standard for the diagnosis of EHE. The lungs and liver are the most commonly involved organs [1, 8, 23]. The tumor tissue is composed of cell-rich regions and fibrotic regions. Immunohistochemistry is helpful in differential diagnosis; EHE stains positively for CD31, CD34, and von Willebrand factor [24]. In the present study, the positive rates of CD31 and CD34 were 97.0%, and it was considered diagnostic. CD31 is also known as platelet endothelial cell adhesion molecule-1 and often expressed in vascular endothelial cells. CD34 is a highly glycosylated transmembrane glycoprotein and associated with the origin of vascular tumors. These may be the reasons why they have high positive rates in EHE. For the diagnosis of tumors arising from soft tissues, particularly in patients with normal tumor markers, the evaluation of these immunohistochemical indicators should be included in routine practice to improve the diagnosis rate.

The differential diagnosis for EHE is broad, with epithelioid angiosarcoma and metastatic signet-ring cell adenocarcinoma being the two most closely similar diseases. In addition to immunohistochemical characteristics, histologic and cytological features are also important in differential diagnosis. Intracytoplasmic vacuoles, stromal changes, and intranuclear inclusions are helpful in differentiating EHE from epithelioid angiosarcoma as the latter is usually composed of solid and sheets aggregates of atypical neoplastic cells [25]. Cytoplasmic vacuoles containing mucin may be found in neoplastic cells of signet-ring cell adenocarcinoma, and this may lead to a misdiagnosis. In cases presenting with such features, erythrocytes within the cytoplasmic vacuoles is an important factor for the diagnosis of EHE [24].

The prognosis of EHE is superior to that of angiosarcoma. In the present study, the 1-, 3-, and 5-year cumulative survival rates were 96.2%, 87.0%, and 75.3%, respectively. One possible reason for such high survival rates is that majority of lesions are located in the superficial tissue, and thus complete resection can be easily achieved.

For the patients who were followed up, the rates of metastases and unresectability of survivors are significantly lower than those who died. This indicates that early and complete resection is beneficial for improving prognosis. Some previous studies reported that risk factors for worse outcomes included weight loss, anemia, hemoptysis, hemorrhagic pleural effusions, and metastases at presentation [26, 27]. Because EHE has higher potential for metastases than other HEs, it has been considered as an authentic angiosarcoma by some authors [3, 28].

There are some limitations of this study. First, because of its retrospective nature, the registration information, patient volume, and variables assessed could not be designed beforehand. Second, due to its rarity, the sample size is small. Third, the site of tumor involvement varied, and thus it was difficult to compare the diagnostic and treatment modalities used.

## Conclusions

EHE is a rare malignant vascular tumor that could occur in any site of the body and is more common in women than in men. EHE has no characteristic symptom, and most patients are asymptomatic and diagnosed incidentally. Moreover, EHE has no specific laboratory and imaging characteristics. Pathology is the gold standard for diagnosis and immunohistochemistry can be helpful. Surgery is the first choice of treatment, and the overall prognosis is acceptable. Metastases and unresectability are associated with poor prognosis.

## Abbreviations

EHE: epithelioid hemangioendothelioma
HE: hemangioendothelioma
CT: computed tomography
MRI: magnetic resonance imaging
